# A case study of an influenza vaccination program for health care workers in Vietnam

**DOI:** 10.1186/s12913-020-05663-y

**Published:** 2020-08-24

**Authors:** Nga T. Ha, Thoa T. M. Nguyen, Tung X. Nguyen, Phu D. Tran, Hang M. Nguyen, Van T. Ha, Kathryn E. Lafond, Jane F. Seward, Jeffrey W. McFarland, Susan Y. Chu

**Affiliations:** 1 Influenza Division, U.S. Centers for Disease Control and Prevention, Hanoi, Vietnam; 2 General Department of Preventive Medicine, Ministry of Health, Hanoi, Vietnam; 3 U.S. Centers for Disease Control and Prevention, Atlanta, GA USA; 4 Task Force for Global Health, Atlanta, GA USA

**Keywords:** Influenza vaccine, Health care workers, Immunization

## Abstract

**Background:**

In 2017, the Vietnam Ministry of Health conducted a demonstration project to introduce seasonal influenza vaccination to health care workers. A total of 11,000 doses of influenza vaccine, single-dose prefilled syringes, were provided free to HCWs at 29 selected hospitals, clinics, and research institutes in four provinces: Hanoi, Khanh Hoa, Dak Lak and Ho Chi Minh City.

**Methods:**

Before the campaign, a workshop was organized to discuss an implementation plan including technical requirements, cold chain, uptake reporting, and surveillance for adverse events following immunization. All sites distributed communication materials and encouraged their staff to register for vaccination. Following immunization sessions, sites sent reports on uptake and adverse events following immunization. Left-over vaccine was transferred to other sites to maximize vaccine use.

**Results:**

The average uptake was 57% for all health care workers, with 11 sites achieving 90% and above. These 11 sites were small with less than 500 staff, including 5 primary hospitals, 3 preventive medicine units, and 2 referral hospitals. Among the six biggest sites with over 1000 staff, four sites had the lowest uptake (14–47%). Most of the high-uptake sites were from the central to the south; only one site, a referral hospital, was from the north. After redistribution of left-over vaccine, only 130 vaccine doses (1.2%) were not used and destroyed. Based on factors that affected uptake, including registration levels, differing communication strategies, availability of vaccination, and commitment by health facility leaders, we recommended ways to increase health care worker coverage; recommendations to improve reporting adverse events following immunization were also made.

**Conclusions:**

The project demonstrated that it was feasible to conduct influenza vaccination campaigns among health care workers in Vietnam. Improvements in promotion of registration, more intense pre-planning, especially at larger facilities, and wider, more consistent availability of communication materials will result in increased efficiency and coverage in this program’s future expansion.

## Background

Seasonal influenza vaccination can reduce morbidity and mortality from influenza disease and has been shown to be a safe, effective intervention in many, mostly developed countries [1–3]. In Vietnam, reported influenza-like illness affects 1.6 to 1.8 million persons per year and the country has experienced two major outbreaks of novel influenza viruses with pandemic potential, the first in 2003 with highly pathogenic influenza A/H5N1 virus and the other in 2009 with influenza A/H1N1pdm09 virus [4]. As of July 2018, three trivalent influenza vaccines were licensed and imported into Vietnam, that include both Northern and Southern hemisphere formulations. Annually, about 700,000 doses are imported; even if all those doses are used, less than 1% of the population in Vietnam is vaccinated with influenza vaccine each year [5].

In 2017, the Vietnam Ministry of Health (MOH) conducted a demonstration project to introduce seasonal influenza vaccination among health care workers. Eleven thousand doses of vaccine were provided for health care workers (HCWs) at selected hospitals, clinics, and research institutes. HCWs are a priority group for influenza vaccination for several reasons. First, they may be at increased risk of contracting influenza at work. Second, influenza in this group may lead to nosocomial outbreaks, especially in immunocompromised patients. Third, available evidence suggests that influenza vaccination of HCWs may provide a protective effect for inpatients in health care settings [6, 7]. Fourth, health care workers are key influencers and potential role models for influenza vaccine acceptance among other target groups [8, 9]. Last, health care workers are an accessible target population that can be reached through their employment sites [10].

The Vietnam MOH recognized the higher risk of influenza infection in this target group in the 2011 guidelines for seasonal influenza diagnosis and treatment, and recommended them as a target group for annual influenza vaccination in the 2013 plan on production and use of influenza vaccine [11, 12]. However, this demonstration project is the first major effort to implement this recommendation and increase vaccine uptake in this important group. This paper presents the demonstration project as a case study with its process, results, key findings and recommendations for further expansion of influenza vaccination in Vietnam.

## Methods

### Project timeline and selection of health facilities

The immunization campaign was conducted from January to May 2017 in selected health units. Criteria for selecting these sites were that the health facilities (i) were willing and supportive of influenza vaccination; (ii) were representative of the region; and (iii) had staff committed to implementing the project. In this demonstration project, health care workers were defined as all medical and non-medical staff of health facilities including permanent and contract personnel. Immunization was on voluntary basis for health units as well as health care workers.

According to the original plan, ten hospitals and preventive medicine centers were selected in four cities/provinces, one in each of the four medical regions, North, Central, Central Highlands and South. They are listed below:

- National Hospital for Tropical Diseases (Hanoi),
- Bach Mai Hospital (Hanoi),
- National Hospital for Obstetrics and Gynecology (Hanoi),
- National Institute of Hygiene and Epidemiology (Hanoi),
- Khanh Hoa General Hospital (Khanh Hoa)
- Dak Lak General Hospital (Dak Lak),
- Cho Ray Hospital (Ho Chi Minh City),
- Tu Du Hospital (Ho Chi Minh City),
- Hospital for Tropical Diseases of HCM City (Ho Chi Minh City),
- Pasteur Institute - HCM City (Ho Chi Minh City)

Of the ten sites, four sites had certified trained immunizers; these staff conducted the influenza immunization sessions at both their own facilities and the other six sites.

### Preparatory activities

In preparation for the campaign, a workshop on planning for the immunization program was conducted with all participating sites to discuss the implementation plan, logistic preparations, and administrative and technical requirements in the campaign. A critical activity for preparation was determining how many health care workers at each health facility were willing to get immunization, who were required to sign up in a registration list. Most of the sites announced the vaccination campaign through briefings, posted the information on the facility’s staff website, and/or sent notifications and registration forms by email or hard copy to all departments. At each site, a focal person was assigned to collect and consolidate lists of registered health care workers from all departments. Upon having the full registration list, sites informed MOH of the quantity of vaccines they needed.

### Vaccines

Vaccine for this project, funded by the Cooperative Agreement IP000821 between U.S. CDC and the General Department of Preventive Medicine (GDPM), was Influvac, single-dose pre-filled syringe influenza vaccine manufactured by Abbott Biologicals B.V., Netherlands containing the northern hemisphere 16/17 formulation with an expiry date of June 30, 2017 [13]**.**

#### Vaccine transportation and storage plan

MOH had Phuc Thien Pharmaceutical Joint Stock Company distribute the 11,000 vaccine doses to the sites. The cold chain was maintained with temperature checks during storage, delivery, and receipt, which were documented. The cold chain system of the National Extended Program for Immunization was in place at two sites; other sites used their own cold chain system.

On a vaccination session day, vaccines were transported from cold storage to immunization sites in standardized cold boxes as per regulations. Unused vaccine at the end of the session was returned to cold storage. After each site completed their vaccinations, the vaccination team made a final record of vaccine used and any unused doses were later reallocated to other sites. The reallocation prioritized sites in the same province to minimize transportation costs. It was reported by all sites that the storage of influenza vaccines for this demonstration project did not cause any changes or impacts to the storage plan of other vaccines or the overall plan of the existing cold chain systems.

### Immunization sessions

Vaccination teams were set up to carry out immunization; each team included one officer to list vaccine recipients and distribute vaccination certificates after immunization, one physician to screen participants, and one vaccinator. Smaller sites used one team, the largest sites used three teams, but most commonly, two teams were established for the immunization sessions. To avoid overcrowding, different departments came for immunization at different times. All sites reported that the arrangement for the campaign did not significantly affect their routine professional work. The vaccination implementation were in line with Government of Vietnam’s policies on immunization in Decree 104/2016/NĐ-CP issued by the Government of Vietnam on July 1, 2016 [14], and Ministry of Health’s guidance including Circular 12/2014/TT-BYT dated March 20, 2014 on management and use of vaccine in immunization [15], Decision 1730/QĐ-BYT dated May 16, 2014 on storage of vaccine [16], Decision No.1731/QĐ-BYT dated May 16, 2014 on arragement of immunization sessions [17], three regulations on monitoring of adverse events following immunization namely Decision 1830/QĐ-BYT dated May 26, 2014 [18], Decision 2535/QĐ-BYT dated July 10, 2014 [19], and Decision 2228/QĐ-BYT dated June 11, 2015 [20].

### Reporting and monitoring of immunization

All immunization sites were required to send a daily summary after each vaccination session and a final report by the end of the immunization campaign. The daily summary included a list of the day’s vaccine recipients and any AEFI cases with appropriate details. AEFIs were monitored for 30 min at the immunization site or were reported to the immunization hotline within 24 h after vaccinations, with daily summaries compiled into an all-site database. These requirements are in consistent with Ministry of Health’s reporting requirements for routine immunization activities.

To ensure the timeliness and quality of reporting, each site assigned one person to consolidate daily the list of vaccine recipients, and coordinate the number of vaccines needed, used and remaining. If the number of health care workers vaccinated was lower than expected, reminders were sent out to registered HCWs; and the site also made extra efforts to encourage HCWs to come for vaccination. This helped the site to reach as many registered HCWs as possible, and even reached HCWs who had not registered for vaccination. The timeliness of daily summaries and final reports from sites to MOH made transfer of left-over vaccines to other sites possible, greatly reducing vaccine wastage.

MOH sent staff to all sites to provide technical support and monitor activities. U.S.CDC joined MOH to monitor at some immunization sites. The monitoring and technical oversight helped identify drawbacks and implement timely adjustments.

### Communication campaign

At each site, registered HCWs were provided a written summary about influenza and influenza vaccine. Some site leaders organized all-hands meetings where they advocated for the influenza vaccination campaign, highlighted the importance of vaccine and encouraged staff to get immunized. During the vaccination session, vaccination staff advised HCWs on the benefits of influenza vaccine and its possible side effects. After getting the shot, each health care worker got a certificate of immunization (each site designed its own certificate). However, because all communication materials developed by MOH for this campaign were not completed before the vaccination started, most sites noted a lack of adequate communication materials, resulting in some HCWs not understanding the purpose of the vaccination campaign and the benefits of influenza immunization, and not registering or coming for vaccination.

## Results

Ten sites were selected at the beginning of the project. However, during implementation, some large sites, such as Bach Mai and Cho Ray Hospitals, had a lower than expected number of health care workers register for vaccination. This resulted in a considerable number of left-over vaccine doses. For this reason, an additional 19 sites in the same four provinces/cities were added to utilize the remaining vaccine. Criteria for selection of additional sites were that the health facilities (i) must be located in the four selected provinces, and (ii) were willing to be part of this demonstration project.

The additional 19 sites are listed below:

- 1 site in Hanoi: General Department of Preventive Medicine (GDPM)
- 1 site in Ho Chi Minh City: Hung Vuong Hospital, Preventive Medicine Center
- 10 sites in Khanh Hoa: Khanh Hoa Hospital of Tropical Diseases, Khanh Hoa Preventive Medicine Center, Pasteur Institute - Nha Trang, Khanh Hoa Health Department, Ninh Hoa District General Hospital, Cam Ranh General Hospital, Hospital of Tuberculosis and Lung Diseases in Khanh Hoa, Health Center of Nha Trang City, Van Ninh District Health Center,
- 7 sites in Dak Lak: Dak Lak Preventive Medicine Center, General Hospital Thien Hanh, General Hospital of Buon Ma Thuot City, Buon Ma Thuot Health Center, General Hospital of Buon Ho district, Buon Ho Health Center, and Tay Nguyen Institute of Hygiene and Epidemiology

Thus, the vaccination campaign was eventually conducted in 29 sites in four provinces.

### Vaccine used

Average uptake of this vaccination program was 57% of all HCWs, with 11 sites achieving 90% and above. These 11 sites were small with less than 500 staff, which included 5 primary hospitals, 3 preventive medicine units, and 2 referral hospitals. Most of them were from the central to the south; only one site was from the north, this was a referral hospital. Among the six biggest sites with over 1000 staff, four sites had the lowest uptake, from 14 to 47%.

Among those HCWs who registered, the average proportion of vaccine recipients were 87%, which was relatively high. At some sites, the number of vaccinated staff exceeded the number of registered staff, reflecting the availability of doses for all HCWs wanting to be vaccinated.

In Hanoi, leftover vaccines were allocated to GDPM in Hanoi and other sites in Dak Lak and Khanh Hoa provinces. Left-over vaccines in Ho Chi Minh City, Dak Lak and Khanh Hoa were transferred to other sites in the same province. The vaccine storage, transportation and delivery were done strictly according to regulations. Only 130 of 11,000 (1.2%) vaccine doses were not used before the expiry date; these 130 doses were destroyed (Table 1).

**Table 1:** Coverage of influenza vaccination at 29 sites of the 2017 demonstration project

No.	Name of sites	Number of staff	Number of registered people	Number of immunized people	% immunized per registered	Uptake rate (% immunized per all staff)
1	Buon Ho District Health Center	75	50	75	150%	100%
2	Ho Chi Minh City Provincial Preventive Medicine Center	170	167	170	102%	100%
3	Pasteur Institute of Nha Trang	170	169	170	101%	100%
4	Khanh Hoa Provincial Preventive Medicine Center	60	60	60	100%	100%
5	Khanh Hoa Hospital of Tropical Diseases	130	130	130	100%	100%
6	Van Ninh District Health Center	164	164	164	100%	100%
7	Buon Ma Thuot City Health Center	50	50	50	100%	100%
8	Pasteur Institute of Ho Chi Minh City	370	370	370	100%	100%
9	Buon Ho District General Hospital	300	300	295	98%	98%
10	National Hospital of Tropical Diseases	480	470	455	97%	95%
11	Dak Lak Provincial Preventive Medicine Center	67	60	60	100%	90%
12	General Department of Preventive Medicine	79	70	70	100%	89%
13	Dak Lak General Hospital	1264	1186	1095	92%	87%
14	Khanh Hoa General Hospital	1400	1203	1205	100%	86%
15	Tay Nguyen Institute of Hygiene and Epidemiology	138	114	116	102%	84%
16	Buon Ma Thuot City General Hospital	300	300	252	84%	84%
17	Khanh Hoa Department of Health	60	60	50	83%	83%
18	Hung Vuong Hospital	750	750	620	83%	83%
19	Thien Hanh Hospital	500	500	395	79%	79%
20	Nha Trang City Health Center	430	300	334	111%	78%
21	Ninh Hoa Regional General Hospital	291	300	214	71%	74%
22	National Institute of Hygiene and Epidemiology	337	262	240	92%	71%
23	Ho Chi Minh City Hospital of Tropical Diseases	690	521	475	91%	69%
24	Cam Ranh Regional General Hospital	300	300	173	58%	58%
25	Tu Du Hospital	2500	1185	1185	100%	47%
26	Khanh Hoa Hospital of Lung and Tuberculosis Diseases	100	100	38	38%	38%
27	Bach Mai Hospital	2790	1500	1043	70%	37%
28	Cho Ray Hospital	3800	1560	1199	77%	32%
29	National Hospital of Obstetrics and Gynecology	1170	350	167	48%	14%
	**TOTAL**	**18,906**	**12,551**	**10,870**	**87%**	**57%**

### Adverse events following immunization

Vaccination sites had trained staff and necessary equipment and medicines to treat acute severe AEFIs immediately and appropriately. However, most HCWs would not stay at the vaccination site for the full 30 min, with some saying they could monitor themselves and return if anything went wrong. There were 38 AEFI cases reported from 7 sites, including pain at the injection site, mild fever, cough, or dizziness. All reported AEFIs were mild and resolved in a few days. There were no severe AEFI cases (Table 2).

**Table 2:** Adverse events following influenza vaccination at the 2017 demonstration project

No.	Name of sites	No. of immunized people	No. of AEFI cases
1	National Hospital of Obstetric and Gynecology	167	1 mild case
2	Khanh Hoa General Hospital	1205	1 mild case
3	Regional General Hospital of Cam Ranh	173	1 mild case
4	Dak Lak General Hospital	1095	1 mild case
5	Buon Ho District Health Clinic	75	2 mild cases
6	Ho Chi Minh City Hospital of Tropical Diseases	475	30 mild cases: sore at injection site
7	Cho Ray Hospital	1199	2 mild cases

## Discussion

There are a number of findings from this demonstration projects, and we make recommendations to improve the coverage and efficiency of the proposed immunization program for HCWs in Vietnam. First, the project had very low wastage rate of vaccines. The use of single dose prefilled syringes in this demonstration project helped ensure lower wastage than is typical of other vaccine presentations, particularly multi-dose vaccine vials [21]. In addition, timely redistribution of unused vaccine to additional health facilities further reduced wastage. However, frequent redistribution of vaccine doses is not ideal for future program planning, and the key findings from this demonstration project in projecting site-level uptake can inform improvements in Vietnam’s influenza immunization program for health care workers.

Second, registration by HCWs in most sites improved vaccination coverage. This was a more important factor in the larger sites, as the six largest sites each with more than 1000 staff had lower registration rates (54%) than the rate for all remaining sites (86%). In future campaigns, greater promotion of the registration process and closer timing of the registration process to the vaccination session dates are recommended. A system should be developed for reminding HCWs, both registered and unregistered, of the dates and times of the vaccination sessions.

Third, sites where leaders actively advocated for influenza immunization and strongly encouraged their staff to get vaccinated had higher uptake of vaccine. Therefore, hospitals are recommended to obtain leaders strong active commitment to the immunization activity.

Forth, overall coverage was lower among health facilities with larger staffs (> 500 HCWs). In addition to the lower registration rates in larger facilities, it is possible that increased workloads at larger sites made vaccination sessions more difficult for HCWs to prioritize and attend. The exceptions to this finding were the two tropical diseases hospitals, which specialize in infectious diseases, that had better uptake, perhaps due to greater awareness of their occupational influenza exposure risk. For similar campaigns in the future, earlier and more intense planning at larger sites and specialized health facilities are critical to ensure sufficient time for registration, communication and preparation for immunization. Management should ensure that HCWs have the time and opportunity to attend vaccination sessions, especially in more work-intensive settings.

Lower coverage in large sites could also be the result of insufficient communication materials and fewer opportunities for face-to-face advocacy efforts, limiting HCWs’ understanding about the campaign and the benefits of influenza vaccination. The lack of information about the campaign, also raises potential ethical concerns about the health and safety of HCWs in these facilities. First, some employees may receive the vaccine without understanding the potential limitations and/or risks of vaccination, although influenza vaccine has repeatedly been shown to be safe and effective. Second, HCWs who were not contacted or did not have access to communication materials may not have been aware of the opportunity to be vaccinated. As communication is essential to improve seasonal influenza vaccine uptake [22] and to allay employee’s concerns, communication materials should be made available well in advance of and face-to-face events should occur before registration opportunities and both should also be made available in advance of vaccination sessions.

Fifth, it is possible that AEFIs, especially mild reactions, were underreported. Many HCWs did not stay for the entire 30 min post vaccination monitoring time. Only seven of 29 sites reported any AEFIs and only one site, Ho Chi Minh City Hospital of Tropical Diseases reported more than 2 AEFIs. No serious AEFIs were reported, but because the program was conducted among HCWs in hospitals or clinics, it was unlikely serious AEFIs were missed. If similar programs are organized, it is recommended that hospitals ensure HCWs be aware of AEFIs, emphasizing the rarity of serious reactions while also stressing the importance for AEFI surveillance.

Last but not least, the storage of vaccines for this demonstration project did not affect the storage plan of other vaccines or the overall plan of the existing cold chain systems, given that the quantity of vaccine in the project were not a significantly large number. For a more extensive immunization program in the future, it’s critical to examine if the existing cold chain is capable of storing extra larger amount of vaccines as part of the planning process. The expansion of the cold chain should be considered, if needed, to ensure vaccine is stored in appropriate conditions.

In addition, experiences and lessons learnt from this influenza vaccination demonstration project, particularly in planning, logistic arrangements and vaccine administration, are also useful for the planning and preparation of vaccination for any emerging diseases including COVID-19, when the vaccine becomes available. Our findings are particularly relevant in case the Vietnam MOH intends to prioritize front-line healthcare workers for COVID-19 vaccination.

This study has a number of limitations. Results of the project, particularly the relatively high uptake rates, may be biased given that the project was conducted in selected facilities that participated in the program on a voluntary basis. On the other hand, all four provinces were big provinces including some larger cities, therefore, experiences and lessons learnt from the project may not be generalizable for a nation-wide program. A bigger campaign in a broader range of provinces would be able to provide a better perception of a program at a bigger scale. Another challenge was that results of the program were mainly dependent on reports by the health facilities; supervision and cross-checking was limited due to reduced number of MOH site visits. We recommend that the MOH and regional level health agencies enhance the level of support and oversight during future efforts.

Future plans include a qualitative study on acceptability of influenza vaccination among HCWs a prospective study on vaccine effectiveness in healthcare workers and cost effectiveness of influenza vaccination in healthcare workers.

## Conclusion

The project demonstrated that it was feasible to conduct an influenza vaccination campaign among HCWs in Vietnam. Expansion of this program to more HCWs at more sites is likely achievable. Key findings and recommendations from this project could improve coverage and efficiency of delivery in future influenza immunization efforts in HCWs.

## Data Availability

All data generated or analyzed during this study are presented in the manuscript.

## Abbreviations

AEFI: Adverse event following immunization
GDPM: General department of preventive medicine
HCW: Healthcare worker
MOH: Ministry of health
U.S. CDC: Centers for diseases control and prevention

## References

[1] Breteler JK, Tam JS, Jit M, Ket JC, De Boer MR (2013). Efficacy and effectiveness of seasonal and pandemic a (H1N1) 2009 influenza vaccines in low and middle income countries: a systematic review and meta-analysis. Vaccine.

[2] Andrew MK, Shinde V, Hatchette T, Ambrose A, Boivin G, Bowie W (2017). Influenza vaccine effectiveness against influenza-related hospitalization during a season with mixed outbreaks of four influenza viruses: a test-negative case-control study in adults in Canada. BMC Infect Dis.

[3] World Health Organization. 2017 [2018 january 1]. *Vaccine use* http://www.who.int/influenza/vaccines/use/en/. Accessed 16 December 2019.

[4] Vietnam Ministry of Health. Health Statistics yearbook 2015. 2017.

[5] World Health Organization. *Made in Viet Nam vaccines: efforts to develop sustainable in-country manufacturing for seasonal and pandemic influenza vaccines: consultation held in Vietnam, April–June 2016*. 2017.

[6] Pavia AT (2010). Mandate to protect patients from health care-associated influenza. Clin Infect Dis.

[7] Poland GA, Tosh P, Jacobson RM (2005). Requiring influenza vaccination for health care workers: seven truths we must accept. Vaccine.

[8] Ditsungnoen D, Greenbaum A, Praphasiri P, Dawood FS, Thompson MG, Yoocharoen P (2016). Knowledge, attitudes and beliefs related to seasonal influenza vaccine among pregnant women in Thailand. Vaccine.

[9] Song Y, Zhang T, Chen L, Yi B, Hao X, Zhou S (2017). Increasing seasonal influenza vaccination among high risk groups in China: do community healthcare workers have a role to play?. Vaccine.

[10] Advisory Committee on Immunization Practices, Centers for Disease Control and Prevention (2011). Immunization of health-care personnel: recommendations of the Advisory Committee on Immunization Practices (ACIP). MMWR Recomm Rep.

[11] Vietnam Ministry of Health. *Decision No.2078/QĐ-BYT dated 23/6/2011 of Minister of Health regarding Guidelines of Seasonal influenza diagnose and treatment*. 2011.

[12] Vietnam Ministry of Health. *Decision No.1950/QĐ-BYT dated 6/6/2013 of Minister of Health regarding Plan for development and use of influenza vaccines*. 2013.

[13] Abbott. Influvac® 2016/2017. Package leaflet: *Information for the user. Suspension for injection in prefilled syringe. Influenza vaccine (surface antigen, inactivated) 2016/2017 season.* 2016.

[14] Government of Vietnam. *Decree 104/2016/NĐ-CP dated July 1st, 2016 by July 1, 2016 guiding about immunization activities*. 2016.

[15] Vietnam Ministry of Health. *Circular 12/2014/TT-BYT dated March 20, 2014 guiding the management and use of vaccine in immunization*. 2014.

[16] Vietnam Ministry of Health, *Decision 1730/QĐ-BYT dated May 16, 2014 guiding the storage of vaccine.* 2014.

[17] Vietnam Ministry of Health. *Decision No.1731/QĐ-BYT dated May 16, 2014 guiding the arrangement of an immunization session*. 2014.

[18] Vietnam Ministry of Health. *Decision 1830/QĐ-BYT dated May 26, 2014 guiding the monitoring, investigation, analysis and assessment of causes of adverse events following immunization.* 2014.

[19] Vietnam Ministry of Health*Decision 2535/QĐ-BYT dated July 10, 2014 guiding the monitoring, care and management of adverse events following immunization.* 2014.

[20] Vietnam Ministry of Health. *Decision 2228/QĐ-BYT dated June 11, 2015 on amendment of Articles 3.1 and 3.3 of the guidelines on monitoring, investigation, analysis and assessment of causes of adverse events following immunization, issued in Decision* 1830/QĐ-BYT*dated May 26, 2014 by Ministry of Health*. 2014.

[21] Drain PK, Nelson CM, Lloyd JS (2003). Single-dose versus multi-dose vaccine vials for immunization programmes in developing countries. Bull World Health Organ.

[22] MacDonald L, Cairns G. Kathryn Angus & Marisa de Andrade (2013) promotional communications for influenza vaccination: a systematic review. J Health Commun. 2013. 10.1080/10810730.2013.840697. 10.1080/10810730.2013.840697 24298886

